# The mechanism of *Leonuri Herba* in improving polycystic ovary syndrome was analyzed based on network pharmacology and molecular docking

**DOI:** 10.3389/jpps.2023.11234

**Published:** 2023-02-15

**Authors:** Mali Wu, Hua Liu, Jie Zhang, Fangfang Dai, Yiping Gong, Yanxiang Cheng

**Affiliations:** ^1^ Department of Obstetrics and Gynecology, Renmin Hospital of Wuhan University, Wuhan, China; ^2^ Department of Breast Surgery, Renmin Hospital of Wuhan University, Wuhan, China

**Keywords:** *Leonuri Herba*, polycystic ovary syndrome, network pharmacology, molecular docking, endocrine disorder

## Abstract

**Background:** Polycystic ovarian syndrome (PCOS) is the most common endocrine disorder affecting women. Chinese herbs have been considered as an alternative treatment for PCOS, and Yi-mu-cao (*Leonuri Herba*) is one of the most commonly used herbs to treat PCOS, which can relieve symptoms of PCOS patients. But the mechanism of its treatment remains unclear.

**Method:** The main active ingredients and potential targets of *Leonuri Herba* were obtained by TCMSP and Swiss Target Forecast, and the related targets of PCOS were obtained by searching DrugBank, GeneCard and DisGeNet databases. The Protein-Protein Interaction (PPI) network was constructed using STRING database. GO and KEGG were used to detect the enrichment pathways of key targets. Cytoscape software was used to construct the component-target-pathway network, analyze the PPI network core, and verify the reliability of target binding by molecular docking technology.

**Result:** 8 components and 116 targets of *Leonuri Herba* on PCOS were screened. Common targets mainly involve the Lipid and atherosclerosis, Endocrine resistance, AGE-RAGE signaling in diabetic complications and other signaling pathways. It is suggested that it can form multi-target and multi-pathway regulatory network through quercetin, kaempferol and other active substances to regulate endocrine disorders and reduce inflammatory response, so as to systematically improve PCOS. Molecular docking experiments showed that the active constituents of Leonurus had good binding activity with potential targets of PCOS.

**Conclusion:** In summary, this study elucidates the potential effect of *Leonuri Herba* on PCOS, which is helpful to provide reference for clinical practice. This is also conducive to the secondary development of motherwort and its monomer components, and precision medicine for PCOS.

## Introduction

Polycystic ovary syndrome (PCOS) can affect 5–18% of women (1). It is characterized by androgen excess, infertility, irregular menstrual cycle, and abnormal ovarian androgen production caused by PCOM (2). PCOS increases the risk of infertility, endometrial dysfunction, cardiovascular disease, diabetes, metabolic syndrome, and other diseases (3). It seriously affected the quality of life of patients. At present, PCOS treatment mainly relies on antiandrogen drugs, insulin sensitizers, ovulation promoting drugs, oral contraceptives and so on (4–6). However, the treatment of PCOS is still a difficult problem in obstetrics and gynecology. As PCOS is a multi-system disease with complex pathological mechanism, heterogeneous symptoms and numerous complications, sometimes western medicines cannot achieve good therapeutic effects, and more drug targets need to be explored.

Yi-mu-cao (*Leonuri Herba*) is naturally found in plants and has traditionally been used in China for thousands of years for uterine contractions, postpartum congestion, breast tenderness, and other gynecological disorders (7, 8). It has been reported as a prescription single herb with antioxidant activity that can treat dysmenorrhea by relieving uterine spasms, reducing inflammation, reducing concentrations of prostaglandin F2α and prostaglandin synthase-2 in uterine smooth muscle, increasing serum progesterone levels, and effectively relieving symptoms of PCOS (9–11). However, because *Leonuri Herba* is a kind of herbal medicine with diverse ingredients and targets, the therapeutic mechanism is not clear at present, and it is necessary to further explore its therapeutic mechanism.

In recent years, Chinese herbal medicine has become a new research hotspot because of its multi-component and multi-target characteristics. But the mechanism of treatment is complex and unclear.

**FIGURE 1:**
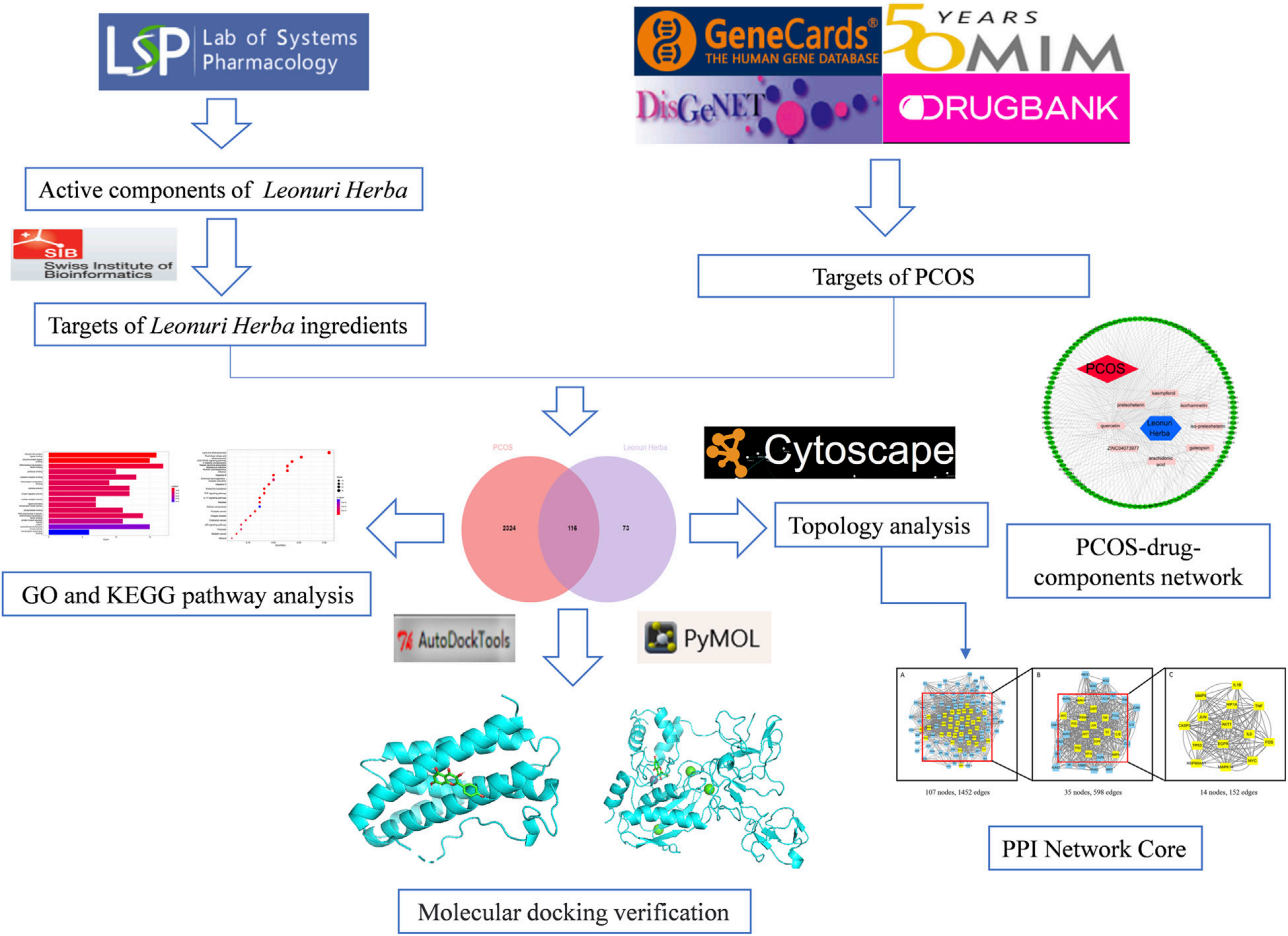
The workflow of the current network pharmacology study.

In this study, we applied a network pharmacology approach to achieve a multilevel study to determine the interaction between motherwort and PCOS. Network pharmacology is a new strategy to study the interaction between drugs and diseases (12). This research method can bring a lot of benefits to traditional Chinese medicine (TCM), because the underlying mechanism of a large proportion of TCM has not been fully understood (13). After network pharmacological analysis, we further confirmed the potential pharmacological effects of *Leonuri Herba* components on PCOS by molecular docking of the analyzed core genes with the main effective drugs. The entire study can be seen in Figure 1.

## Materials and methods

### Screening and target analysis of active constituents from *Leonuri Herba*

The active components were found by TCMSP (http://tcmspw.com/tcmsp.php), which were screened according to the two ADME attribute values of oral availability (oral bio-availability, OB) ≥30% and drug-like drugs (drug-likeness, DL) ≥ 0.18 (14). A total of 8 active components were analyzed in Table 1. In addition, using the PubChem website (https://pubchem.ncbi.nlm.nih.gov/), get PubChem ID, smiles number, and 2D chemical structures. Then, SwissTargetPrediction (http://swisstargetprediction.ch/) was used to predict protein targets, the feasibility of value as the >0.5, get the target of 189. All target genes are converted into gene symbols using the UniProt knowledge base (http://www.UniProt.org).

**TABLE 1:** A list of the active compounds in *Leonuri Herba*.

Mol ID	Molecule name	ОВ (%)	DL
MOL001418	galeopsin	61.01548	0.3753
MOL001420	ZINC04073977	37.99619	0.75755
MOL001421	preleoheterin	85.97259	0.33044
MOL001422	Iso-preleoheterin	66.28878	0.33032
MOL000098	quercetin	46.43335	0.27525
MOL001439	arachidonic acid	45.57325	0.20409
MOL000354	isorhamnetin	49.60438	0.306
MOL000422	kaempferol	41.88225	0.24066

### Collection of disease targets for PCOS

**FIGURE 2:**
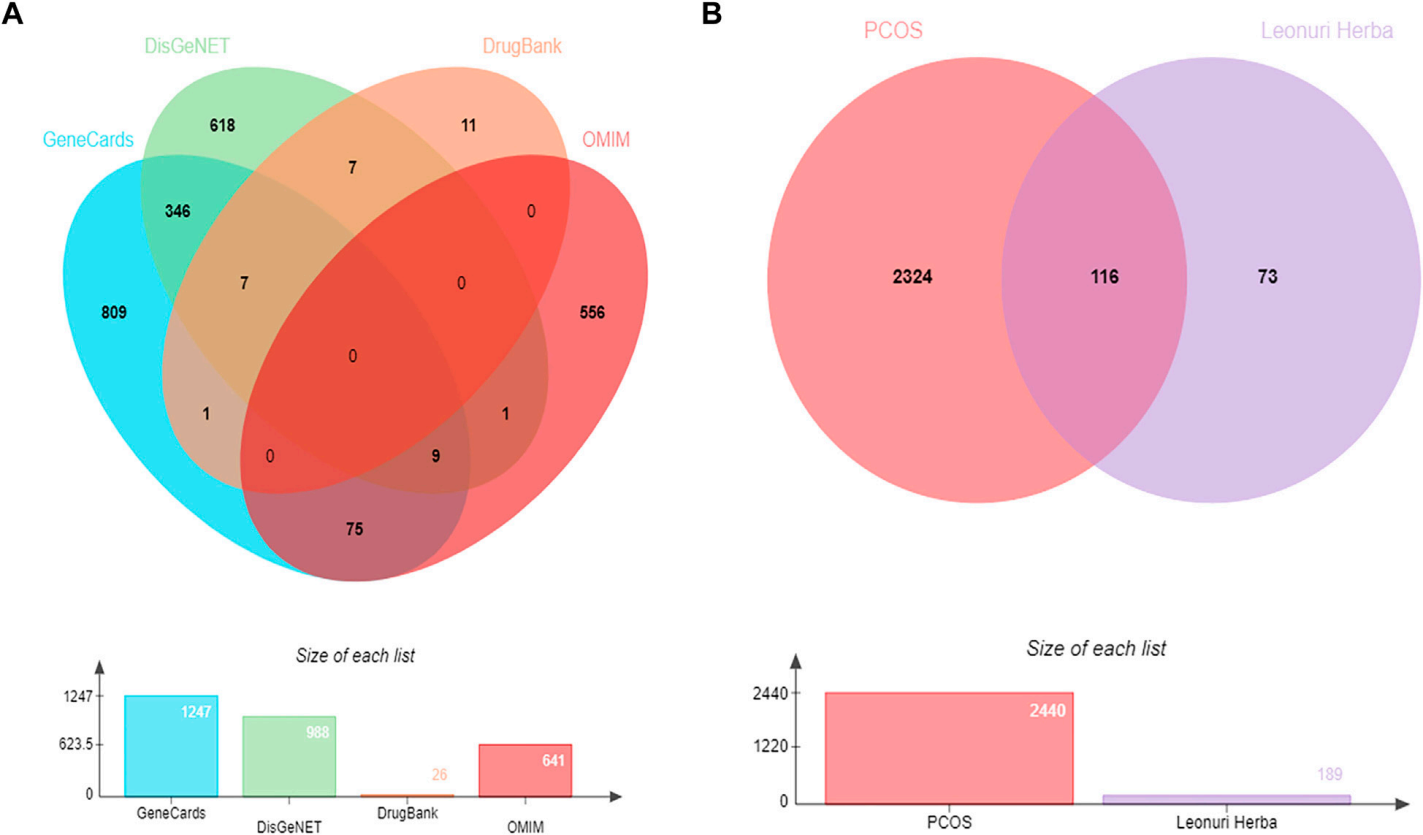
**(A)** Venn diagram of disease targets in four databases. **(B)** Venn diagram of active ingredients and disease targets. PCOS, polycystic ovarian syndrome.

With “PCOS” and “Polycystic ovary syndrome” as key words, Mine Gene in GeneCard database (https://www. genecards.org) and Map database of OMIM (http://www.omim.org), DisGeNet database (http://bidd.nus.edu.sg/group/cjttd) the potential targets of PCOS, Enter DrugBank database (https://www.drugbank.ca) to search for first-line clinical western drug targets for the treatment of PCOS. In the GeneCards database, a higher score indicates that the target is closely related to the disease. According to experience, if there are too many targets, the target whose Score is greater than the median is set as the potential target of PCOS twice. After merging the four disease database targets together, there are 2,440 disease target intersection genes in the four databases in Figure 2A.

### Construction and analysis of disease-medicine network

To explore active ingredients of *Leonuri Herba* targets with PCOS disease targets, the online mapping tool platform (http://www.bioinformatics.com.cn/) was used to draw the Venn diagram, get 116 intersection genes in Figure 2B. Cytoscape3.7.2 software was used to map the TCM-active ingredients-disease target network.

### Constructing PPI network of intersection targets between PCOS and *Leonuri Herba*

The STRING database (https://string-db.org) was used to construct the PPI network diagram of 116 related targets: Set the biological species as “*Homo sapiens*,” and the minimum interaction threshold Highest confidence >0.7 as the screening condition. Each node represents a protein and its structure, and each edge represents the association between different proteins.

### Screening key targets

The PPI results are exported as TSV files and then imported into Cytoscape3.7.2 for further network analysis using the CytoNCA plug-in (15). Each gene receives a score based on the six dimensions of the “Betweenness/Closeness/Degree/Eigenvector/LAC/Network.” 14 key targets were screened out under the condition that the median score was more than 6 parameters.

### GO and KEGG pathway enrichment analyses

The results of pathway enrichment analysis from Gene Ontology (GO) and the Kyoto Encyclopedia of Genes and Genomes (KEGG, https://www.kegg.jp/) were applied to the STRING online database (https://string-db.org/) to annotate and classify common targets (16). After setting an adjusted P value cutoff of 0.05, we collected and analyzed the data by Rstudio 3.6.3 (Bioconductor, clusterProfiler).

### Molecular docking between *Leonuri Herba* and key targets

According to the enrichment results of compounds KEGG and GO and the comprehensive analysis of the current research status, we selected the two most critical molecules of this drug: quercetin (MOL000098) and kaempferol (MOL000422). TCMSP database (https://tcmspw.com/tcmsp.php) to download the molecular structures and transform them into mol2 formats. The structure of the receptor can be downloaded from the PDB Protein Database (http://www.rcsb.org). The docking simulation was performed with selected key proteins such as AKT1, IL6, EGFR, and MMP9 by AutoDock Vina 1.5.6. The binding affinity between molecules and proteins is predicted based on the docking minimum free energy. The lower the free energy, the higher the affinity. The results are saved in the pdbqt file. Finally, the results were analyzed and demonstrated by PyMOL.

## Results

### Identification of the ingredients of *Leonuri Herba* and predicted target genes of PCOS

There was a total of 8 active compounds of *Leonuri Herba*, as shown in Table 1. 189 potential targets could be obtained after prediction and deweighting of the active compounds with potential targets through screening in SwissTargetPrediction database. The PCOS related target genes were downloaded from four disease databases, and the genes obtained from GeneCards, DisGeNET, OMIM and DrugBank were screened and de-weighted to obtain 2,440 genes. They were then combined with 189 target genes from *Leonuri Herba* for analysis. Finally, 116 common target genes were extracted. Venn diagrams were plotted accordingly in Figure 2.

### Construction and analysis of target PPI network

**FIGURE 3:**
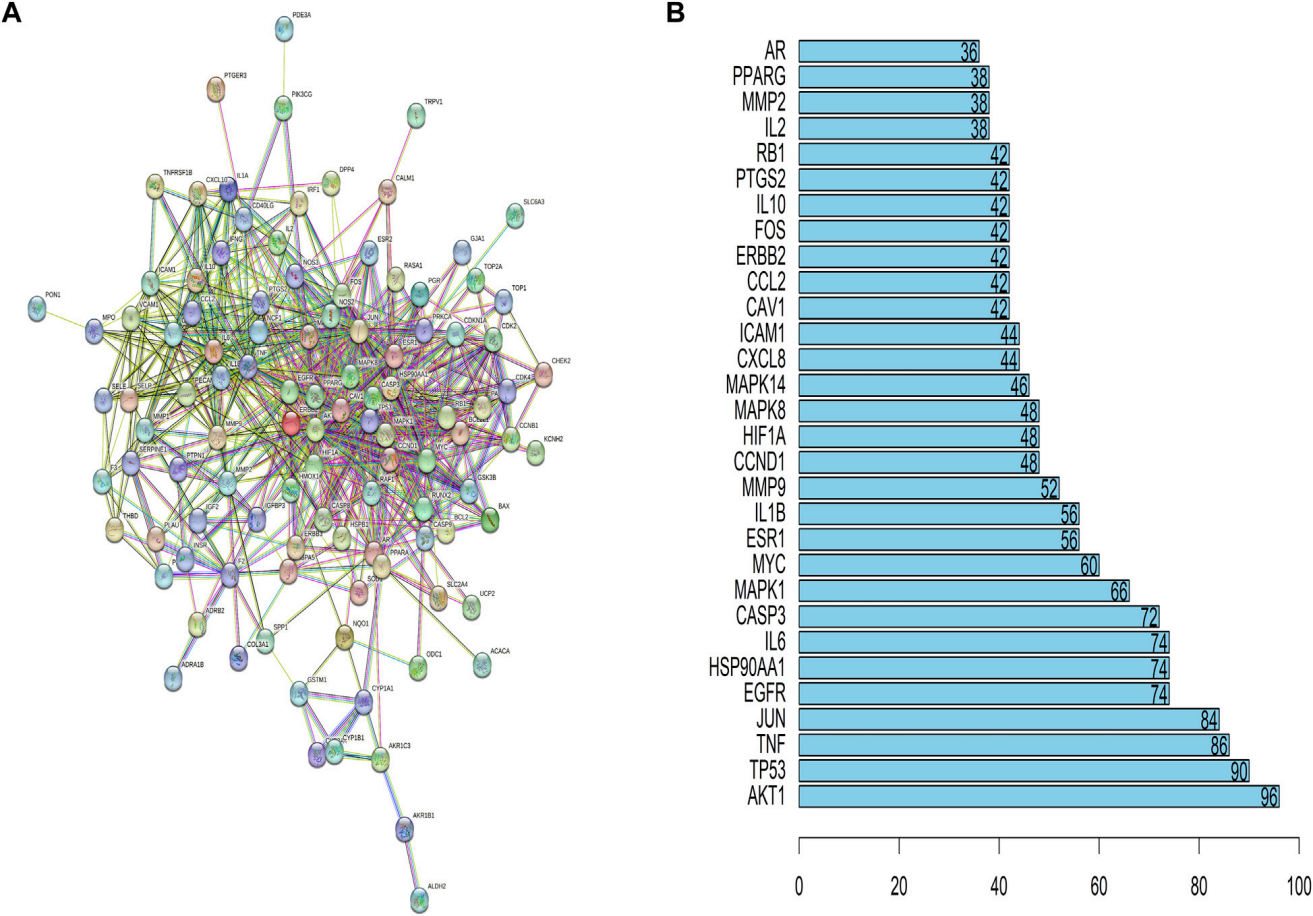
**(A)** Protein–protein interaction network of the common active ingredients and disease targets. 122 nodes (target genes) and 726 edges (associations between proteins) are presented. **(B)** Top 30 PPI network core gene visualization.

Target genes were uploaded to STRING online database to form PPI network. 116 nodes (genes) and 726 edges (interactions) were identified, representing the major genes corresponding to the active ingredient of *Leonuri Herba* in Figure 3. The more interacting target genes are located in the central region of the network. RAC-alpha serine/threonine-protein kinase (AKT1), interleukin-6 (IL6), epidermal growth factor receptor (EGFR), vascular endothelial growth factor A (VEGFA), matrix metalloproteinase-9 (MMP9), transcription factor Jun (JUN), myc proto-oncogene protein (MYC), interleukin-1 beta (IL1B), and hypoxia-inducible factor 1-alpha (HIF1A) are most important genes in *Leonuri Herba* ‘s pharmacological effects on PCOS according to their degree.

### The PPI network core of *Leonuri Herba* and PCOS cross targets

**FIGURE 4:**
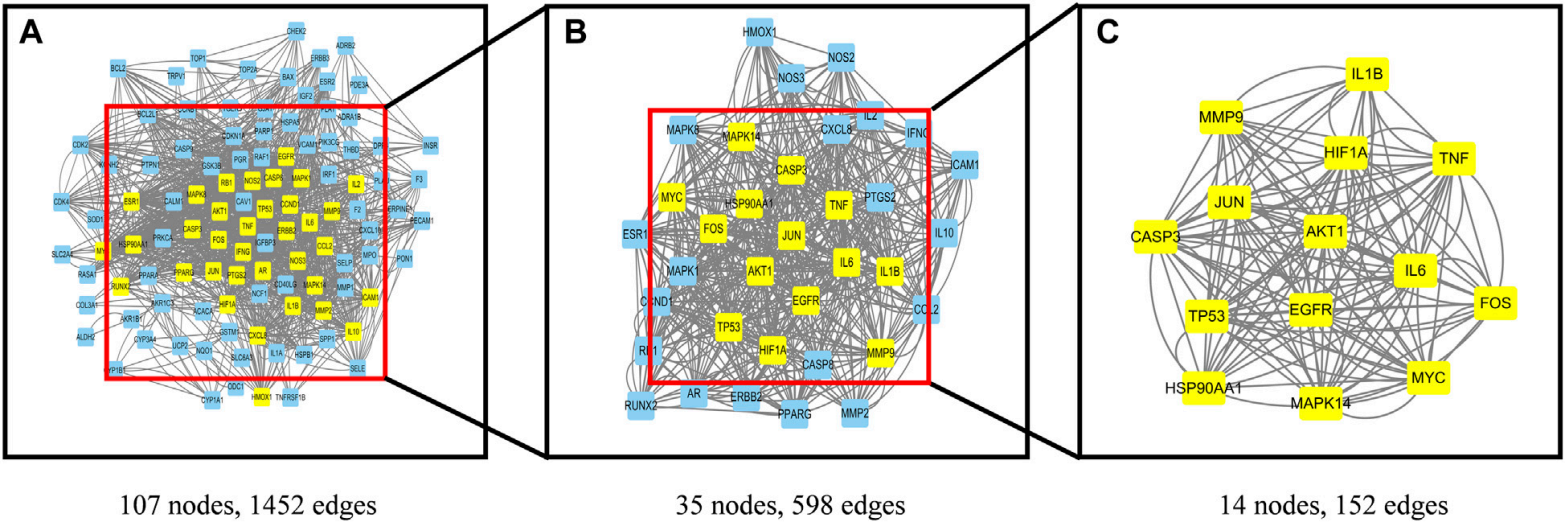
Topological analysis of the PPI network. CytoNCA score greater than median. The yellow squares represent the core genes for screening. The blue squares represent unscreened genes. **(A)** The PPI network of PCOS-Leonuri Herba target genes. **(B)** The PPI network of significant proteins extracted from **(A)**. **(C)** The PPI network of crucial Leonuri Herba targets for PCOS treatment extracted from **(B)**.

The PPI results are exported as TSV files and then imported into Cytoscape3.7.2 for further network analysis using the CytoNCA plug-in. Through 6 parameters in CytoNCA “Betweenness/Closeness/Degree/Eigenvector/LAC/Network” to filter 116 genes. First filter condition, “Betweenness: 49.38088469/Closeness: 0.436213992/Degree: 24/Eigenvector: 0.063172609/LAC: 11.38461538/Network: 12.8”. Getting 35 nodes, 598 edges; Second filter condition, “Betweenness: 11.15689699/Closeness: 0.653846154/Degree: 32/Eigenvector: 0.156082243/LAC: 20.66666667/Network: 23.41193154”. Get 14 nodes (core genes), 152 edges, specific genes such as JUN, AKT1, HSP90AA1, CASP3, FOS, MYC, EGFR, HIF1A, TP53, TNF, IL6, IL1B, MMP9, MAPK14 in Figure 4. Betweenness/Closeness/Degree/Eigenvector/LAC/Network of the 14 core genes in Supplementary Figure S1.

### Biological functional analysis

**FIGURE 5:**
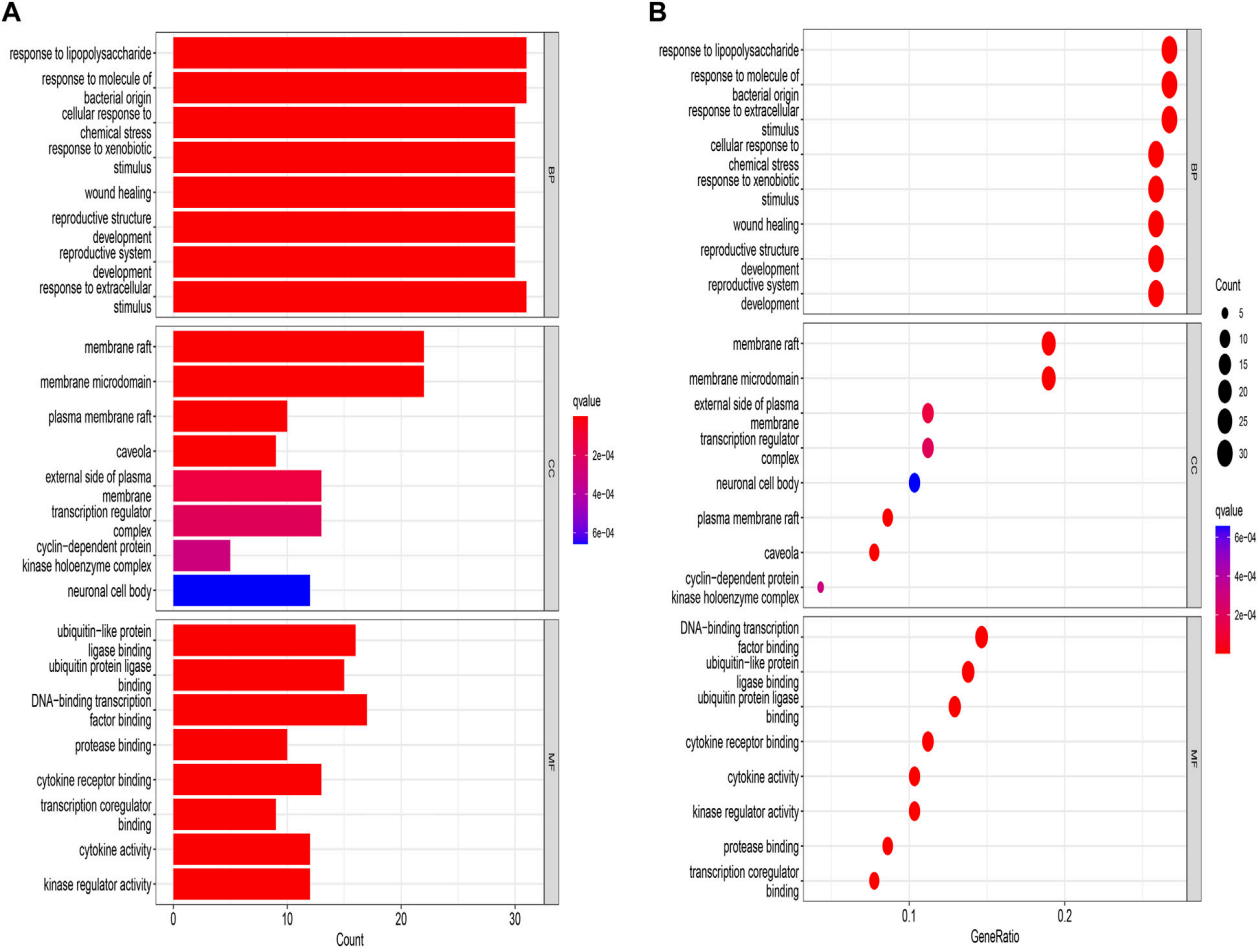
GO Pathway Enrichment Analysis. **(A)** The horizontal axis of BP, CC, and MF bar represents the number of genes enriched in each, while the color visualizes the significance based on the corrected P value. **(B)** The bubble diagram demonstrates the gene proportion enriched in each subset. BP, biological process; MF, molecular function; CC, cell component.

**FIGURE 6:**
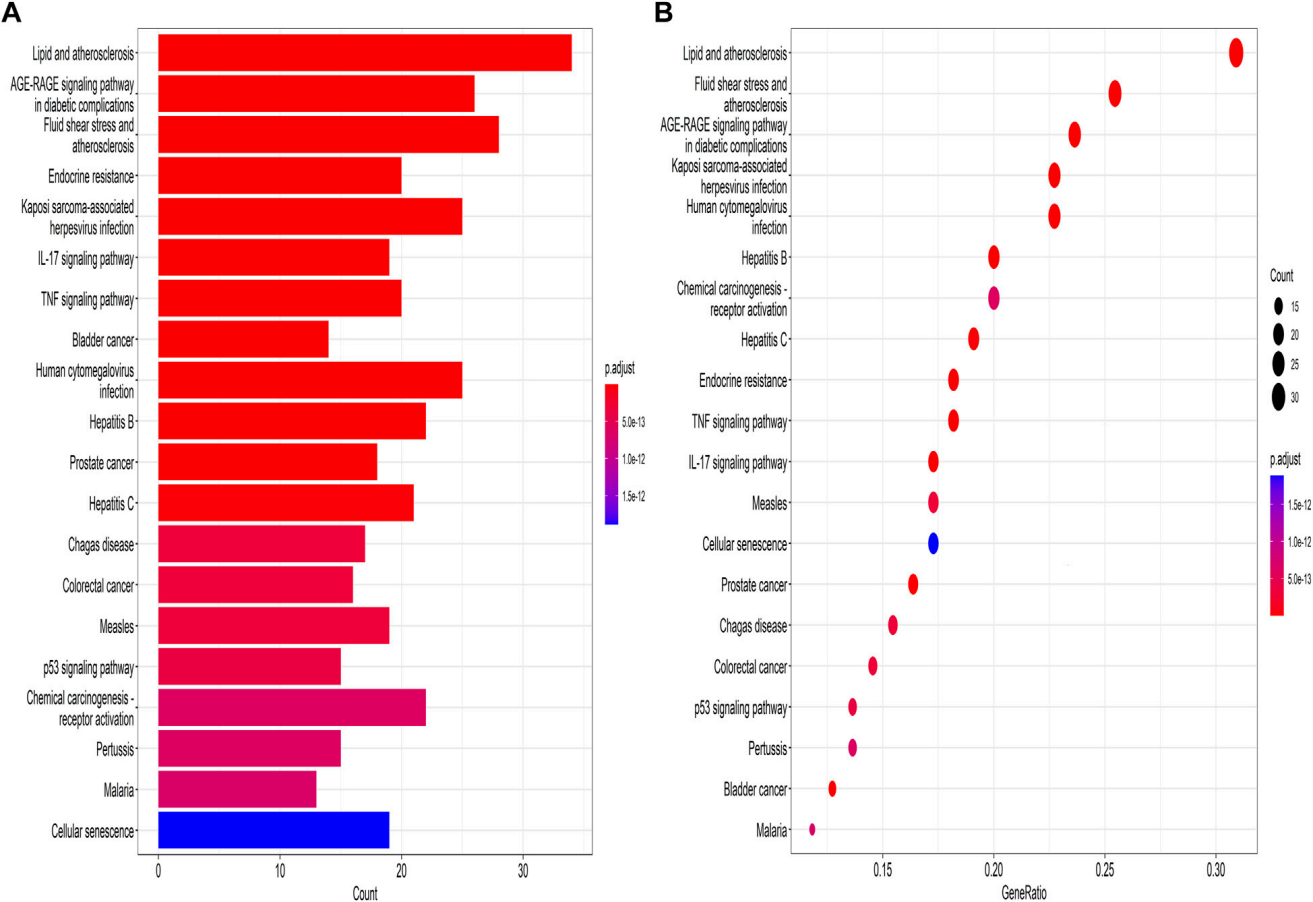
KEGG pathway enrichment analysis. **(A)** The red color in the upper part represents greater significance, while the blue represents less significance according to corrected P value. **(B)** The bubble diagram demonstrates the gene proportion enriched in each entry.

Subsequently, GO enrichment analysis was performed. The top 8 enrichment results for BP, MFs and CCs are listed in Figure 5. The results suggest that biological processes include cellular responses to lipopolysaccharide, reproductive phylogeny, oxidative stress, and reactive oxygen species. In the drug-disease interaction, the molecular function is manifested by high level of nuclear steroid receptor activity, protein serine/threonine/tyrosine kinase activity, DNA binding and transcription factor binding, and the interaction was mainly enriched in membrane raft and membrane microdomain. The related pathway of *Leonuri Herba* was obtained through KEGG enrichment analysis. 166 signaling pathways were discovered, and the top 20 were shown in Figure 6. Lipid and atherosclerosis (has05417), AGE-RAGE signaling pathway (hsa04933) and fluid shear stress and atherosclerhass (hsa05418) are most prominent in the bar graph of Figure 6A.

### Construction of compound-target-disease pathway

**FIGURE 7:**
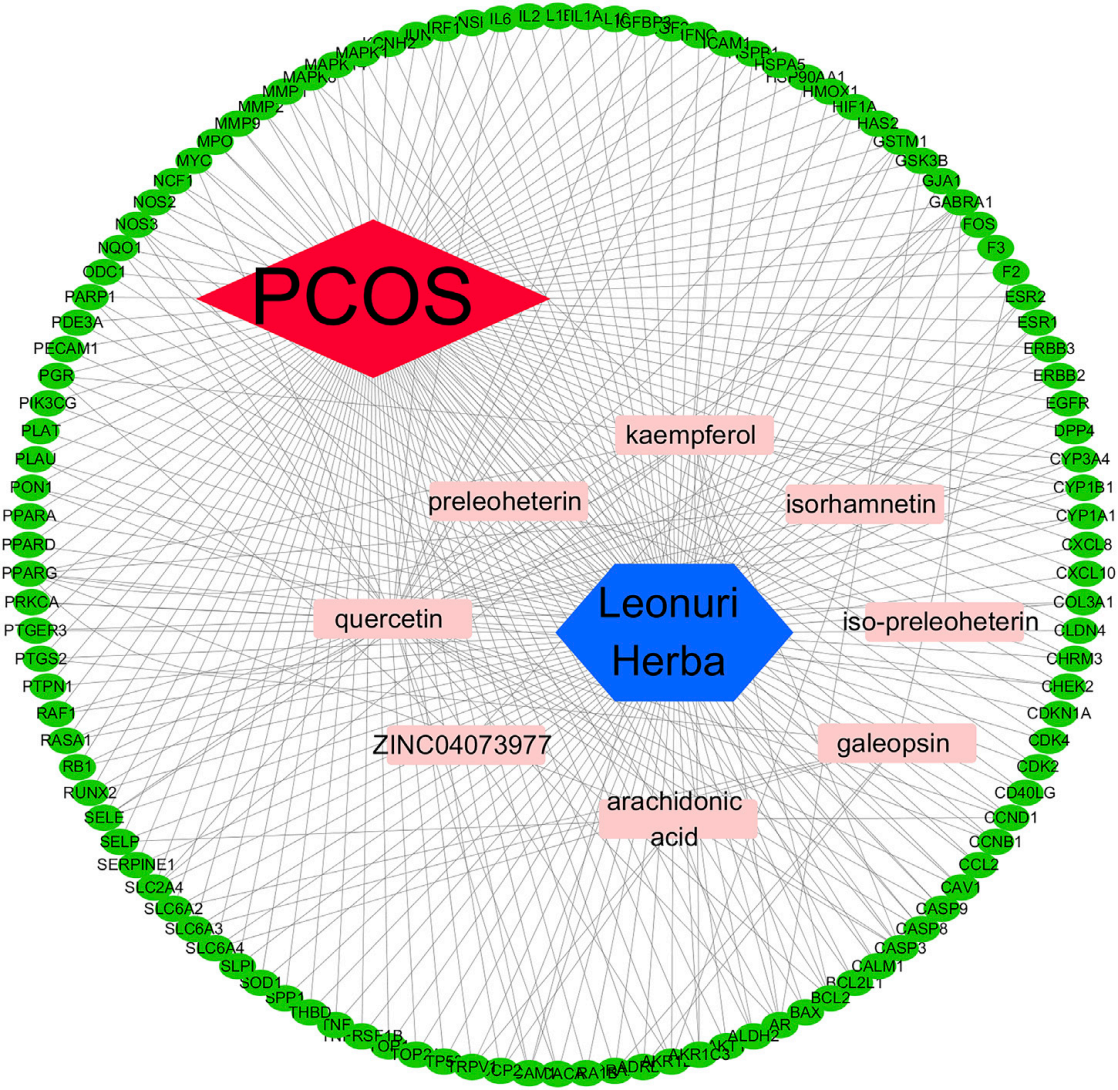
Chinese medicine-active ingredient-disease target network. PCOS, polycystic ovarian syndrome.

Plot the composition-target-pathway network between PCOS and *Leonuri Herba* (Figure 7). The analysis of Figure 7 shows that *Leonuri Herba* ort may have a therapeutic effect on PCOS through multiple active components, targets, and pathways. Among them, quercetin, isorhamnetin, and kaempferol are important components, and the main targets are AKT1, EGFR, IL6, MMP9 and so on.

### Molecular docking of the compounds and the core targets

**FIGURE 8:**
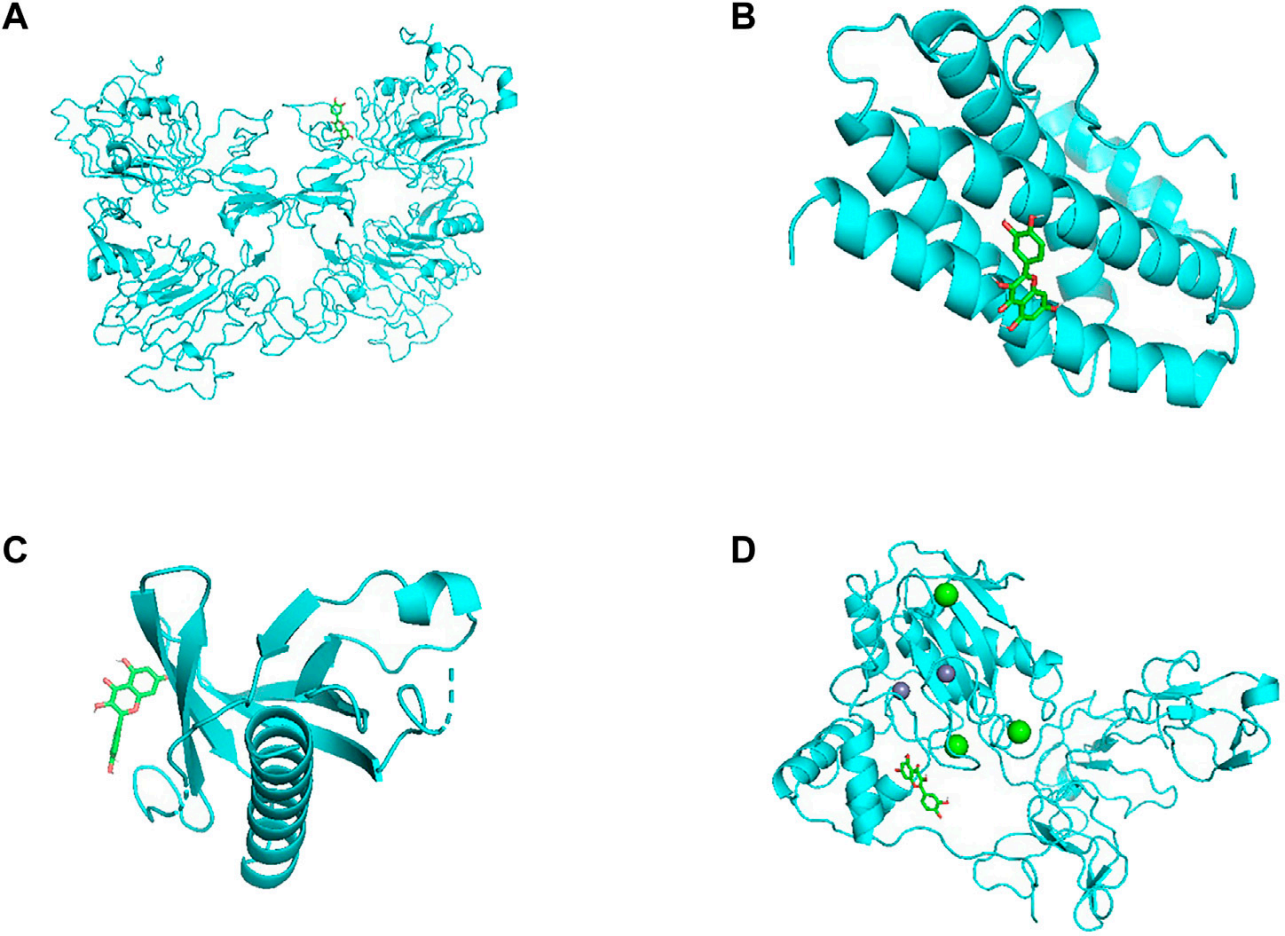
**(A)** Quercetin docked with EGFR. **(B)** Quercetin docked with IL6. **(C)** Quercetin docked with AKT1. **(D)** Quercetin docked with MMP9.

**FIGURE 9:**
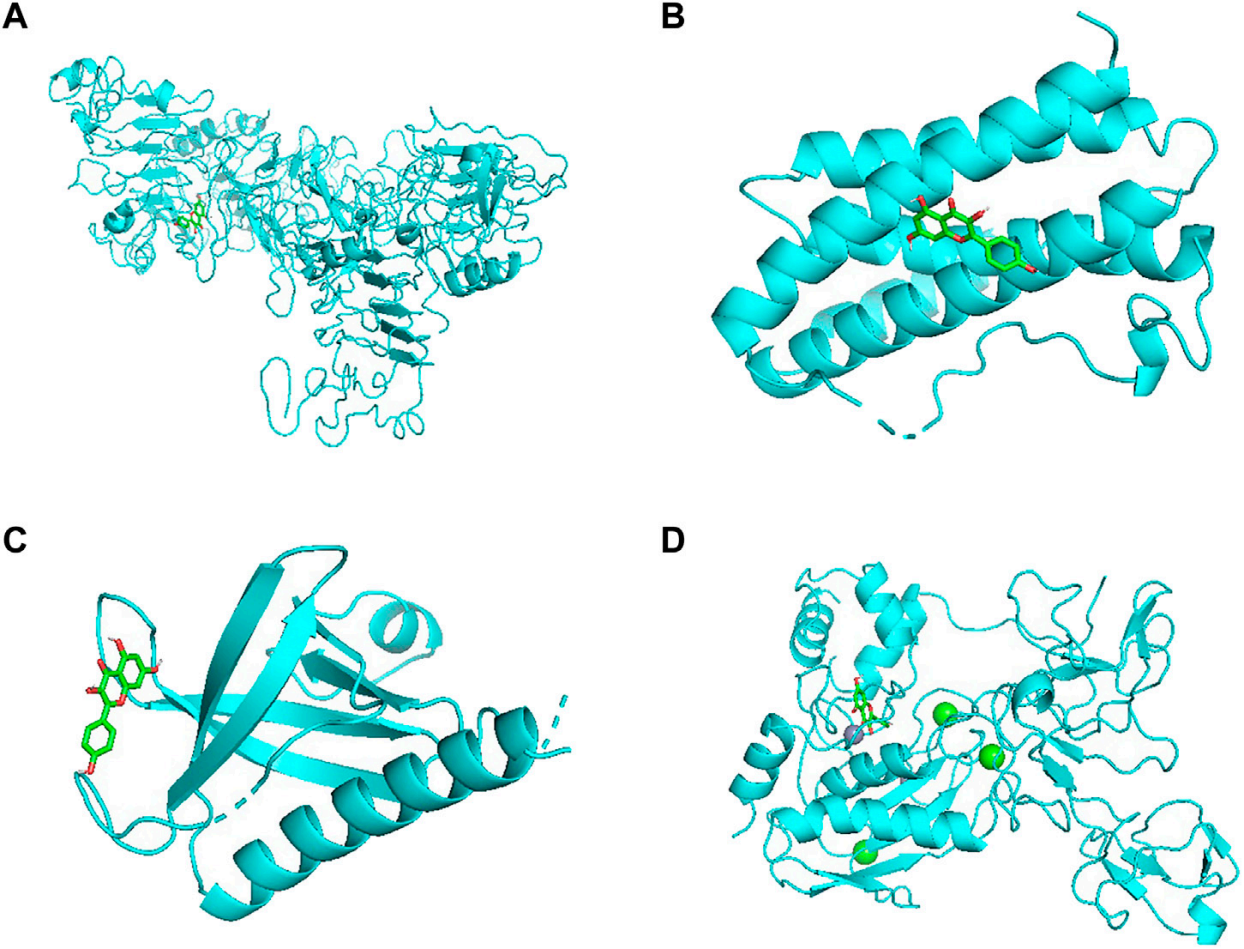
**(A)** Kaempferol docked with EGFR. **(B)** Kaempferol docked with IL6. **(C)** Kaempferol docked with AKT1. **(D)** Kaempferol docked with MMP9.

Molecular docking verification was carried out by AutoDock Vina. The results showed that the minimum free energy of quercetin (MOL000098), kaempferol (MOL000422) and key targets AKT1, IL6, EGFR and MMP9 were shown in Table 2. Especially among all the possible binding structures, kaempferol has the best affinity with MMP9 (−9.6 kcal/mol). Other detailed results are shown in Figures 8, 9.

**TABLE 2:** Affinity of active chemicals to key targets.

Receptor name	Ligand name	Affinity(kcal/mol)
AKT1	quercetin	−6.1
IL6	quercetin	−6.9
EGFR	quercetin	−8.6
MMP9	quercetin	−8.1
AKT1	kaempferol	−6.1
IL6	kaempferol	−6.7
EGFR	kaempferol	−8.5
MMP9	kaempferol	−9.6

## Discussion

PCOS is one of the most common endocrine disorders among women of reproductive age and is heterogeneous in that women may develop reproductive, endocrine, and/or metabolic symptoms that vary throughout their lives (17, 18). Due to its wide range of causes, it can lead to a range of disease symptoms. Such as low fertility, sparse menstruation, hirsutism, and insulin resistance pose a serious threat to women’s reproductive health and daily life (19). However, the current western medicine treatment may be unable to solve parts of the symptoms of PCOS. The Chinese medicine and TCM may make up for the shortcomings of Western medicine.

In recent years, more and more Chinese medicines and their preparations have come into the world’s attention. Of course, these TCMs can also be effective in treating PCOS, and many studies on the treatment of PCOS with TCMs have been reported (20). For example, TCM can be used to treat PCOS with oligomenorrhea and amenorrhea, relieve ovulation dysfunction, obesity, insulin resistance, and improve ovulation and pregnancy rates (21–24).

*Leonuri Herba* in this study is one of the traditional Chinese herbs, which has been widely used to treat gynecological and obstetric diseases for thousands of years. Its main ingredients are reported to include leonurine, 4′,5-dihydroxy-7-methoxyflavone, rutin, hyperoside, apigenin, quercetin, kaempferol and salicylic acid (25). So far, its main components have been found to have antioxidant stress, ROS reduction levels, anti-inflammatory, treatment of infertility or menstrual disorders, treatment of cardiovascular and cerebrovascular diseases (9, 26, 27). However, due to the complex chemical composition and unclear pharmacological mechanism of TCM, it faces great obstacles in pharmacological research, quality control and supervision (28). At present, TCM database and target prediction technology have brought new ideas and strategies for the research on the basis and mechanism of TCM pharmacodynamic substances and helped to identify the advantages of TCM such as good efficacy, high safety, multi-component, and multi-target (29). Network pharmacology allows researchers to study the interaction between the chemical composition of *Leonuri Herba* and PCOS-related genes.

In this study, we used the newly developed bioinformatics technology to explore the possible interaction between *Leonuri Herba* and PCOS in the network. We found that quercetin and kaempferol are the main active components of this drug, which can play an important role in anti-inflammation and antioxidation, which has been effectively verified in molecular docking research. In addition, quercetin, the main ingredient, has been shown to be effective in treating PCOS. It can significantly reduce the expression of testosterone (T), estradiol (E2), luteinizing Hormone (LH), Bax, IL-1β, IL-6 and TNF-α, increase the expression of FSH and Bcl-2, and inhibit the expression of androgen receptor (AR), thus restoring the maturation and ovulation of oocytes (30, 31). Moreover, it can also be anti-inflammatory to improve insulin resistance and relieve PCOS endocrine disruption (32, 33). It has been reported that kaempferol-7-O-methylether, another major component, may increase the activity of PPAR-γ and inhibit the TGF-β pathway, thus improving the metabolic disorder and ovarian fibrosis in PCOS rats (34).

In addition, we screened out 14 core genes (JUN, AKT1, HSP90AA1, CASP3, FOS, MYC, EGFR, HIF1A, TP53, TNF, IL6, IL1B, MMP9, MAPK1). Among them, High levels of AKT1 is associated with Granulosa cells (GC) dysfunction (35). MMP9 has been confirmed to be associated with atherosclerotic thrombosis, endothelial dysfunction, and non-alcoholic fatty liver disease in PCOS patients (36–38). IL1B, IL6 and TNF are associated with PCOS inflammation, endoplasmic reticulum stress and recurrent abortion, and can be regulated by the active components of *Leonuri Herba* (39–44). *Leonuri Herba* regulates the expression of these genes through AGE-RAGE, PI3K-Akt and MAPK signaling pathways.

To the best of our knowledge, this is the first time to reveal the active ingredients of *Leonuri Herba* and their pharmacological effects on PCOS. This helps researchers and pharmacologists understand the mechanisms of motherwort. However, further *in vitro* experiments are needed to verify the predicted process.

## Conclusion

Through the analysis of network pharmacology, the step-by-step mining of data and the analysis of multi-target and multi-way methods, we can more clearly understand the important role of *Leonuri Herba* in PCOS. Our current research is only from the simple prediction of drug components on the target nuclear pathway of network pharmacology. However, further experiments are needed to confirm the specific therapeutic mechanism of *Leonuri Herba* in the treatment of PCOS. Look forward to the accurate treatment of PCOS with active ingredients of TCM in the future.

## Data Availability

The datasets presented in this study can be found in online repositories. The names of the repository/repositories and accession number(s) can be found in the article/Supplementary Material.

## Abbreviation

AR, androgen receptor; AKT1, RAC-alpha serine/threonine-protein kinase; E2, estradiol; EGFR, epidermal growth factor receptor; FSH, follicle stimulating hormone; GO, gene ontology; HIF1A, hypoxia-inducible factor 1-alpha; IL-6, interleukin-6; IL1B, interleukin-1 beta; JUN, transcription factor Jun; KEGG, kyoto encyclopedia of genes and genomes; LH, luteinizing hormone; MAPK, mitogen-activated protein kinase; MMP9, matrix metalloproteinase-9; MYC, Myc proto-oncogene protein; PCOS, polycystic ovary syndrome; PPI, protein-protein interaction; T, testosterone; TCM, Traditional Chinese medicine; TCMSP, Traditional Chinese Medicine System Pharmacology Database and Analysis Platform; VEGFA, vascular endothelial growth factor A.

## References

[1] Joham AE Norman RJ Stener-Victorin E Legro RS Franks S Moran LJ Polycystic ovary syndrome. Lancet Diabetes Endocrinol (2022) 10(9):668–80. 10.1016/s2213-8587(22)00163-2 35934017

[2] Azziz R Carmina E Chen Z Dunaif A Laven JSE Legro RS Polycystic ovary syndrome. Nat Rev Dis Primers (2016) 2:16057. 10.1038/nrdp.2016.57 27510637

[3] Andrade VHLD Mata AMOFD Borges RS Costa-Silva DR Martins LM Ferreira PMP Current aspects of polycystic ovary syndrome: A literature review. Rev Assoc Med Bras (2016) 62(9):867–71. 10.1590/1806-9282.62.09.867 28001262

[4] Tanbo T Mellembakken J Bjercke S Ring E Abyholm T Fedorcsak P. Ovulation induction in polycystic ovary syndrome. Acta Obstet Gynecol Scand (2018) 97(10):1162–7. 10.1111/aogs.13395 29889977

[5] Dokras A Sarwer DB Allison KC Milman L Kris-Etherton PM Kunselman AR Weight loss and lowering androgens predict improvements in health-related quality of life in women with PCOS. J Clin Endocrinol Metab (2016) 101(8):2966–74. 10.1210/jc.2016-1896 27253669 PMC4971336

[6] Oguz SH Yildiz BO. An update on contraception in polycystic ovary syndrome. Endocrinol Metab (Seoul) (2021) 36(2):296–311. 10.3803/enm.2021.958 33853290 PMC8090477

[7] Zhu YZ Wu W Zhu Q Liu X. Discovery of Leonuri and therapeutical applications: From bench to bedside. Pharmacol Ther (2018) 188:26–35. 10.1016/j.pharmthera.2018.01.006 29360539

[8] Miao LL Zhou QM Peng C Liu ZH Xiong L. Leonurus japonicus (Chinese motherwort), an excellent traditional medicine for obstetrical and gynecological diseases: A comprehensive overview. Biomed Pharmacother (2019) 117:109060. 10.1016/j.biopha.2019.109060 31195353

[9] Lin MJ Chen HW Liu PH Cheng WJ Kuo SL Kao MC. The prescription patterns of traditional Chinese medicine for women with polycystic ovary syndrome in taiwan: A nationwide population-based study. Medicine (Baltimore) (2019) 98(24):e15890. 10.1097/md.0000000000015890 31192922 PMC6587642

[10] Zhao B Peng Q Poon EHL Chen F Zhou R Shang G Leonurine promotes the osteoblast differentiation of rat BMSCs by activation of autophagy via the PI3K/Akt/mTOR pathway. Front Bioeng Biotechnol (2021) 9:615191. 10.3389/fbioe.2021.615191 33708763 PMC7940513

[11] Cheng H Bo Y Shen W Tan J Jia Z Xu C Leonurine ameliorates kidney fibrosis via suppressing TGF-β and NF-κB signaling pathway in UUO mice. Int Immunopharmacol (2015) 25(2):406–15. 10.1016/j.intimp.2015.02.023 25727888

[12] Poornima P Kumar JD Zhao Q Blunder M Efferth T. Network pharmacology of cancer: From understanding of complex interactomes to the design of multi-target specific therapeutics from nature. Pharmacol Res (2016) 111:290–302. 10.1016/j.phrs.2016.06.018 27329331

[13] Li S Zhang B. Traditional Chinese medicine network pharmacology: Theory, methodology and application. Chin J Nat Med (2014) 11(2):110–20. 10.3724/sp.j.1009.2013.00110 23787177

[14] Ru J Li P Wang J Zhou W Li B Huang C Tcmsp: A database of systems pharmacology for drug discovery from herbal medicines. J Cheminform (2014) 6:13. 10.1186/1758-2946-6-13 24735618 PMC4001360

[15] Tang Y Li M Wang J Pan Y Wu FX. CytoNCA: A cytoscape plugin for centrality analysis and evaluation of protein interaction networks. Biosystems (2015) 127:67–72. 10.1016/j.biosystems.2014.11.005 25451770

[16] Ogata H Goto S Sato K Fujibuchi W Bono H Kanehisa M. KEGG: Kyoto Encyclopedia of genes and Genomes. Nucleic Acids Res (1999) 27(1):29–34. 10.1093/nar/27.1.29 9847135 PMC148090

[17] Rudnicka E Kunicki M Calik-Ksepka A Suchta K Duszewska A Smolarczyk K Anti-mullerian hormone in pathogenesis, diagnostic and treatment of PCOS. Int J Mol Sci (2021) 22(22):12507. 10.3390/ijms222212507 34830389 PMC8619458

[18] Neven ACH Laven J Teede H Boyle J. A summary on polycystic ovary syndrome: Diagnostic criteria, prevalence, clinical manifestations, and management according to the latest international guidelines. Semin Reprod Med (2018) 36(1):005–12. 10.1055/s-0038-1668085 30189445

[19] Dapas M Dunaif A. Deconstructing a syndrome: Genomic insights into PCOS causal mechanisms and classification. Endocr Rev (2022) 43(6):927–65. 10.1210/endrev/bnac001 35026001 PMC9695127

[20] Liao WT Su CC Lee MT Li CJ Lin CL Chiang JH Integrative Chinese herbal medicine therapy reduced the risk of type 2 diabetes mellitus in patients with polycystic ovary syndrome: A nationwide matched cohort study. J Ethnopharmacol (2019) 243:112091. 10.1016/j.jep.2019.112091 31325604

[21] Lai L Flower A Prescott P Wing T Moore M Lewith G. Standardised versus individualised multiherb Chinese herbal medicine for oligomenorrhoea and amenorrhoea in polycystic ovary syndrome: A randomised feasibility and pilot study in the UK. BMJ Open (2017) 7(2):e011709. 10.1136/bmjopen-2016-011709 PMC5293993 28159846

[22] Moini Jazani A Nasimi Doost Azgomi H Nasimi Doost Azgomi A Nasimi Doost Azgomi R. A comprehensive review of clinical studies with herbal medicine on polycystic ovary syndrome (PCOS). DARU J Pharm Sci (2019) 27(2):863–77. 10.1007/s40199-019-00312-0 PMC6895349 31741280

[23] Ried K. Chinese herbal medicine for female infertility: An updated meta-analysis. Complement Therapies Med (2015) 23(1):116–28. 10.1016/j.ctim.2014.12.004 25637159

[24] Hu J Shi W Xu J Liu S Hu S Fu W Complementary and alternative medicine for the treatment of abnormal endometrial conditions in women with PCOS: A systematic review and meta-analysis of randomized controlled trials. Evid-Based Complement Altern Med (2021) 2021:1–17. 10.1155/2021/5536849 PMC8105096 34012472

[25] Lam KY Wang Y Lam T Ku C Yeung W Zhao Z. Correlation between quality and geographical origins of Leonuri Herba revealed by the qualitative fingerprint profiling and quantitative determination of chemical components. Chin Med (2022) 17(1):46. 10.1186/s13020-022-00592-w 35413864 PMC9003958

[26] Liu X Cao W Qi J Li Q Zhao M Chen Z Leonurine ameliorates adriamycin-induced podocyte injury via suppression of oxidative stress. Free Radic Res (2018) 52(9):952–60. 10.1080/10715762.2018.1500021 30334481

[27] Shi XD Zhang JX Hu XD Zhuang T Lu N Ruan CC. Leonurine attenuates obesity-related vascular dysfunction and inflammation. Antioxidants (Basel) (2022) 11(7):1338. 10.3390/antiox11071338 35883829 PMC9311755

[28] Wang Y Lou XT Shi YH Tong Q Zheng GQ. Erxian decoction, a Chinese herbal formula, for menopausal syndrome: An updated systematic review. J Ethnopharmacol (2019) 234:8–20. 10.1016/j.jep.2019.01.010 30658181

[29] Jiang Y Liu N Zhu S Hu X Chang D Liu J. Elucidation of the mechanisms and molecular targets of yiqi shexue formula for treatment of primary immune thrombocytopenia based on network pharmacology. Front Pharmacol (2019) 10:1136. 10.3389/fphar.2019.01136 31632275 PMC6780007

[30] Zheng S Chen Y Ma M Li M. Mechanism of quercetin on the improvement of ovulation disorder and regulation of ovarian CNP/NPR2 in PCOS model rats. J Formos Med Assoc (2022) 121(6):1081–92. 10.1016/j.jfma.2021.08.015 34538551

[31] Rashidi Z Khosravizadeh Z Talebi A Khodamoradi K Ebrahimi R Amidi F. Overview of biological effects of Quercetin on ovary. Phytother Res (2021) 35(1):33–49. 10.1002/ptr.6750 32557927

[32] Neisy A Zal F Seghatoleslam A Alaee S. Amelioration by quercetin of insulin resistance and uterine GLUT4 and ERα gene expression in rats with polycystic ovary syndrome (PCOS). Reprod Fertil Dev (2019) 31(2):315–23. 10.1071/rd18222 30103849

[33] Pourteymour Fard Tabrizi F Hajizadeh-Sharafabad F Vaezi M Jafari-Vayghan H Alizadeh M Maleki V. Quercetin and polycystic ovary syndrome, current evidence and future directions: A systematic review. J Ovarian Res (2020) 13(1):11. 10.1186/s13048-020-0616-z 32005271 PMC6993490

[34] Zhou Y Lan H Dong Z Li W Qian B Zeng Z Rhamnocitrin attenuates ovarian fibrosis in rats with letrozole-induced experimental polycystic ovary syndrome. Oxid Med Cell Longev (2022) 2022:1–18. 10.1155/2022/5558599 PMC9162838 35663203

[35] Nekoonam S Naji M Nashtaei MS Mortezaee K Koruji M Safdarian L Expression of AKT1 along with AKT2 in granulosa-lutein cells of hyperandrogenic PCOS patients. Arch Gynecol Obstet (2017) 295(4):1041–50. 10.1007/s00404-017-4317-9 28271235

[36] Dambala K Paschou SA Michopoulos A Siasos G Goulis DG Vavilis D Biomarkers of endothelial dysfunction in women with polycystic ovary syndrome. Angiology (2019) 70(9):797–801. 10.1177/0003319719840091 30969784

[37] Gonzalez F Kirwan JP Rote NS Minium J. Glucose ingestion stimulates atherothrombotic inflammation in polycystic ovary syndrome. Am J Physiology-Endocrinology Metab (2013) 304(4):E375–83. 10.1152/ajpendo.00491.2012 PMC3566504 23249695

[38] Chen Y Ma L Ge Z Pan Y Xie L. Key genes associated with non-alcoholic fatty liver disease and polycystic ovary syndrome. Front Mol Biosci (2022) 9:888194. 10.3389/fmolb.2022.888194 35693550 PMC9174783

[39] Moshfegh F Balanejad SZ Shahrokhabady K Attaranzadeh A. Crocus sativus (saffron) petals extract and its active ingredient, anthocyanin improves ovarian dysfunction, regulation of inflammatory genes and antioxidant factors in testosterone-induced PCOS mice. J Ethnopharmacol (2022) 282:114594. 10.1016/j.jep.2021.114594 34480994

[40] Yuan B Luo S Feng L Wang J Mao J Luo B. Resveratrol regulates the inflammation and oxidative stress of granulosa cells in PCOS via targeting TLR2. J Bioenerg Biomembr (2022) 54(4):191–201. 10.1007/s10863-022-09942-7 35836030

[41] Borthakur A D Prabhu Y Valsala Gopalakrishnan A. Role of IL-6 signalling in polycystic ovarian syndrome associated inflammation. J Reprod Immunol (2020) 141:103155. 10.1016/j.jri.2020.103155 32526588

[42] Brenjian S Moini A Yamini N Kashani L Faridmojtahedi M Bahramrezaie M Resveratrol treatment in patients with polycystic ovary syndrome decreased pro-inflammatory and endoplasmic reticulum stress markers. Am J Reprod Immunol (2020) 83(1):e13186. 10.1111/aji.13186 31483910

[43] Alkhuriji AF Al Omar SY Babay ZA El-Khadragy MF Mansour LA Alharbi WG Association of IL-1β, IL-6, TNF-α, and TGFβ1 gene polymorphisms with recurrent spontaneous abortion in polycystic ovary syndrome. Dis Markers (2020) 2020:6076274. 10.1155/2020/6076274 32454906 PMC7232732

[44] Vaez S Parivr K Amidi F Rudbari NH Moini A Amini N Quercetin and polycystic ovary syndrome; inflammation, hormonal parameters and pregnancy outcome: A randomized clinical trial. Am J Reprod Immunol (2022) e13644. 10.1111/aji.13644 36317442

