# Supplementary material for: The mechanism of Leonuri Herba in improving polycystic ovary syndrome was analyzed based on network pharmacology and molecular docking
Source: J Pharm Pharm Sci. 2023 Feb 15;26:11234. doi: 10.3389/jpps.2023.11234 (PMC9990637; doi:10.3389/jpps.2023.11234)
Supplement: Supplementary file 1 [file Image1.pdf]

The main parameters of the top 14 targets in the PPI network

name	Betweenness	Closeness	Degree	Eigenvector	LAC	Network
FOS	0.4	0.764706	18	0.230713	15.11111	16
JUN	4.11746	1	26	0.306928	19.38462	24.68577
AKT1	4.11746	1	26	0.306928	19.38462	24.68577
MYC	1.243651	0.8125	20	0.247962	16	17.35232
EGFR	2.550794	0.928571	24	0.290403	18.66667	21.67068
HIF1A	3.381746	0.928571	24	0.286363	18	21.20203
HSP90AA1	0.2	0.722222	16	0.207754	13.5	14.4
TP53	2.550794	0.928571	24	0.290403	18.66667	21.67068
TNF	2.610317	0.928571	24	0.289761	18.66667	21.73206
IL6	3.298413	0.928571	24	0.286273	18	21.20203
IL1B	0.45	0.722222	16	0.201658	13	13.86667
MMP9	0.5	0.764706	18	0.229264	15.11111	16.18824
CASP3	2.990476	0.928571	24	0.287651	18.33333	21.50585
MAPK14	1.588889	0.8125	20	0.247306	15.6	16.87513

Supplement Figure1 . The main parameters of the top 14 targets in the PPI network.
